# Physiological Changes During Ischemia–Reperfusion Indicating Myocardial Damage Induced by Chronic and Excessive Consumption of *Hibiscus sabdariffa* L.

**DOI:** 10.3390/toxics14080671

**Published:** 2026-07-29

**Authors:** Linaloe Manzano-Pech, Maria Elena Soto, Juan Carlos Torres-Narváez, Raúl Martínez-Memije, Vicente Castrejon-Tellez, Verónica Guarner-Lans, Sara Caballero-Chacón, Félix Leao Rodríguez-Fierros, María de la Luz Ibarra-Lara, Israel Pérez-Torres

**Affiliations:** 1 Department of Physiology and Pharmacology UNAM, Facultad de Medicina y Veterinaria y Zootecnia, Av. Universidad 3000, Coyoacán 04510, Mexico; loe_mana@hotmail.com (L.M.-P.); saracacha@fmvz.unam.mx (S.C.-C.); 2 Research Direction, Instituto Nacional de Cardiología Ignacio Chávez, Juan Badiano 1, Sección XVI, Tlalpan, Mexico City 14080, Mexico; mesoto50@hotmail.com; 3 Department de Pharmacology, Instituto Nacional de Cardiología Ignacio Chávez, Juan Badiano 1, Sección XVI, Tlalpan, Mexico City 14080, Mexico; juancarlostn63@hotmail.com (J.C.T.-N.); luzibarralara@gmail.com (M.d.l.L.I.-L.); 4 Department de Electromechanical Instrumentation, Instituto Nacional de Cardiología Ignacio Chávez, Juan Badiano 1, Sección XVI, Tlalpan, Mexico City 14080, Mexico; raulmmemije@yahoo.com; 5 Department of Physiology, Instituto Nacional de Cardiología Ignacio Chávez, Juan Badiano 1, Sección XVI, Tlalpan, Mexico City 14080, Mexico; vicente.castrejon@cardiologia.org.mx; 6 Sistema Nacional de Investigadores, Secretaria de Ciencias, Humanidades, Tecnología e Innovación, Av. Insurgentes sur 1582, Colonia Crédito Constructor, Benito Juarez, Mexico City 03940, Mexico; gualanv@yahoo.com; 7 Laboratorio de Patología Veterinaria, Facultad de Ciencias Naturales, Universidad Autónoma de Querétaro, Santiago de Querétaro 76230, Mexico; felix.rodriguez@uaq.mx; 8 Department of Cardiovascular Biomedicine, Instituto Nacional de Cardiología Ignacio Chávez, Juan Badiano 1, Sección XVI, Tlalpan, Mexico City 14080, Mexico

**Keywords:** *Hibiscus sabdariffa* L., reductive stress, ischemia–reperfusion, ROS, NrF2

## Abstract

Ingestion of *Hibiscus sabdariffa* L. (HSL) provides antioxidants with beneficial effects for several pathologies with underlying oxidative stress. However, excessive, chronic consumption of the antioxidant-rich diet with HSL in healthy rats may possibly induce reductive stress (RS) with damaging effects. The aim of this study was to evaluate if the consumption of an infusion of 6% HSL for two months alters the function and structure of the myocardium. A total of 24 male Wistar rats were divided into three groups: Control (C), HSL infusion at 6% for two months (HSL 6%), and a washout group that received HSL 6% for two months followed by two months of natural water (HSL ± 6%). Myocardial performance during ischemia–reperfusion (I/R) was evaluated by using the isolated Langendorff heart model, accompanied by surface electrocardiography and histopathological analyses. Chronic 6% HSL intake increased systolic pressure (*p* ≤ 0.03), NrF2 expression, and elevated coronary vascular resistance, while it depressed mechanical performance and led to bradycardia and critical, extended asystolic/sinus pauses. Histopathology showed dense, permanent networks of interstitial and perivascular collagen encapsulating the hypertrophied cardiomyocytes. Chronic 6% HSL infusion was associated with myocardial structural, mechanical, and hemodynamic alterations, and with electrical changes during I/R. The washout group showed possible partial functional differences associated with withdrawal, although interpretation is limited by the longer study period.

## 1. Introduction

The therapeutic value of *Hibiscus sabdariffa* Linnaeus (HSL) is extensively documented. This is owed to its rich concentration of bioactive phytochemicals, including polyphenols, anthocyanins, flavonoids, ascorbic acid, amino acids, and minerals, among others [1]. The administration of HSL infusions at a concentration of 2–3% has consistently demonstrated high efficacy in restoring the cellular redox balance under pathological conditions characterized by oxidative stress (OS), such as metabolic syndrome and essential hypertension in healthy organisms [2]. The exogenous antioxidants collaborate with endogenous enzymatic systems to neutralize reactive oxygen species (ROS) and support physiological homeostasis [3]. However, the conventional assumption that supplementation with a high dose of antioxidants in healthy subjects has arisen.

Prolonged and excessive ingestion of antioxidant-rich diets in healthy states can oversaturate counter-regulatory networks of OS, forcing the cellular environment into a paradoxical condition known as reductive stress (RS) and leading to the loss of redox homeostasis [4]. RS is defined by an overactivation of enzymatic/non-enzymatic antioxidant systems, an increase in total antioxidant capacity (TAC), and the accumulation of reducing equivalents such as nicotinamide adenine dinucleotide/nicotinamide adenine dinucleotide phosphate (NADH/NADPH), reduced glutathione/glutathione disulfide (GSH/GSSG), and oxidized/reduced thiols [5]. This results in a critical depletion of physiological ROS that triggers systemic maladaptive cascades. It damages the role of ROS as second messengers regulating cellular proliferation, mechanical vascular contractility, syncytial communication, spermatogenesis, and ovulation among other cellular pathways [6].

Our research group has systematically explored the multi-organic consequences of RS using a model of chronic consumption of a 6% HSL infusion in healthy Wistar rats. We demonstrated that the excessive intake of this infusion causes systemic alterations, characterized by an upregulated inflammatory profile in plasma, alongside an unexpected elevation in systolic blood pressure (SBP) [7]. At the vascular level, we reported altered aortic vascular reactivity, which is associated with a decrease in glutathione-S-transferase activity and an increase in TAC [8]. Furthermore, our recent work in renal pathology revealed that prolonged consumption of the 6% infusion induces severe kidney damage [9]. This renal impairment is driven by an overproduction of hydrogen sulfide, which elevates cellular reducing power, induces mitochondrial dysfunction via decreased oxidative phosphorylation in complexes I and IV, and results in structural remodeling, indicated by a retraction of the glomerular tuft and tubulointerstitial fibrosis [10].

On the other hand, the myocardium operates as a highly coordinated, functional syncytium in which mechanical contractility and electrical conduction depend on the integrity of the extracellular matrix (ECM), ionic homeostasis, and an uninterrupted supply of ATP. This mechanical syncytium maintains systemic perfusion, making it uniquely sensitive to structural and metabolic remodeling [11]. While the detrimental effects of RS caused by the ingestion of 6% HSL on plasma biomarkers, vascular smooth muscle reactivity, and renal architecture have been established by our group, its specific impact on the heart remains unknown. The question arises whether the systemic inflammatory and hypertensive states induced by chronic ingestion of 6% HSL could extend to the cardiac ECM, or if the effect of this sustained antioxidant overload may affect coronary compliance and electrical coupling. In normal conditions, the collagen of the ECM provides the necessary structural support to withstand systolic stress; however, chronic pro-inflammatory profiles alter its turnover, promoting fibrosis and ventricular stiffness [12]. Cardiac electromechanical coupling also requires the precise synchronization of gap junctions, where connexin 43 (Cx43), the predominant pore-forming protein, is of importance. The assembly and opening of Cx43 is modulated by the redox state, with its cysteine residues being highly susceptible to post-translational modifications [13]. Under normal physiological conditions, basal ROS maintain appropriate Cx43 phosphorylation, gap-junction function, and syncytial communication. Based on previous experimental evidence, it may be hypothesized that excessive accumulation of reducing equivalents could disrupt this balance, causing dephosphorylation or lateralization of Cx43, thus affecting longitudinal conduction velocity and syncytial communication [14]. Since maintaining the electrical ionic gradients of the cardiac syncytium and ECM homeostasis are highly energy-dependent processes, an energy deficit deprives the tissue of the metabolic substrates necessary to sustain its function [15]. Consequently, when faced with hemodynamic challenges such as I/R, a preexisting bioenergetic collapse and electrical uncoupling could alter coronary capacitance, leaving the heart vulnerable to lethal arrhythmias and acute failure.

In this context, the development of cardiac arrhythmias is associated with alterations in tissue excitability and conduction velocity, both of which depend on membrane ion channels, electrochemical gradients, cellular architecture, and intercellular electrical coupling [16]. Gap-junction uncoupling generally slows impulse propagation and disrupts electrical communication between cardiomyocytes. Moderate reductions in intercellular coupling may decrease the electrotonic load imposed on activated cells and increase the safety factor for conduction. However, more severe or heterogeneous uncoupling can markedly slow conduction, promote conduction block, and create a substrate for reentrant arrhythmias [17]. This is due to charge accumulation in the intracellular space of individual cardiomyocytes [18]. However, this increase in intracellular charges leads to faster movement of action potentials across cells but slower movement between them due to cell decoupling [19]. However, other local, genetic, or environmental factors may exist that are integrated within the mechanisms of damage and could lead to structural or hemodynamic functional alterations in the heart. This is further compromised if there is inflammation and/or oxidative or reductive stress, which could have a direct effect on ventricular dysfunction and arrhythmias [20]. Therefore, the aim of the present study was to determine whether chronic and excessive ingestion of a 6% HSL infusion for two months compromises the structural, mechanical, and hemodynamic performance of the myocardium in hearts of healthy Wistar rats subjected to I/R, and whether these effects can be reversed if treatment is suspended.

## 2. Materials and Methods

### 2.1. Preparation of the HSL 6% Infusion

The HSL infusion was prepared by adding 60 g of dried calyces of HSL to 1 L of boiling water. The mixture was maintained at a boil for 10 min, cooled to room temperature, and subsequently filtered. The resulting infusion was stored at 4 °C until administration. A fresh preparation was made weekly to prevent fermentation. It was offered to the rats ad libitum as their drinking fluid.

### 2.2. Determinations of Polyphenols, Total Flavonoids, Total Anthocyanins, and Vitamin C in the HSL 6% Infusion

Total vitamin C in the 6% HSL infusion was quantified according to the method described by Jagota. Briefly, 100 μL of the infusion was mixed with 200 μL of 0.20 mM Folin–Ciocalteu reagent. The reaction mixture was vigorously agitated, incubated for 10 min, and the absorbance was recorded at 760 nm. The vitamin C concentration was calculated from a calibration curve prepared with ascorbic acid as the reference standard [21].

The total flavonoid content in the 6% HSL infusion was measured using the Jia method. This spectrophotometric assay estimates flavanols and flavones, including apigenin, chrysin, luteolin, morin, quercetin, kaempferol, myricetin, and galangin, by measuring absorbance at 510 nm. Quantification was performed using a quercetin calibration curve [22].

Total anthocyanins were determined following the method of Lee, with absorbance measurements obtained at 520 and 700 nm. The results were expressed as cyanidin-3-glucoside equivalents [23].

### 2.3. Calculation for the Sample Size

The mean systolic blood pressure (SBP) in healthy rats was reported to be between 116 mmHg and 112.8 mmHg, with a variance of 19, in rats from the National Institute of Cardiology Ignacio Chavez. Based on this, the sample size per group was estimated by μ (SBP of Wistar rats), with 95% confidence and a maximum error (ME) of 3.2 mmHg, according to the following formula: ME: |μ − x| = |116 − 112.8| = 3.2 mmHg, Confidence: |μ − x| = |116 − 112.8| = 3.2, SS: *n* = ($\sigma_{\bar{x}}$)^2^ × σ^2^/(EM^2^) = (2^2^) × (19)/(3.2)^2^= (4 × 19)/(10.24) = 76/10.24 = 7.42, where ME = the maximum error, $\sigma_{\bar{x}}$ = the number of standard deviations of the mean estimator, σ^2^= the variance in the SBP of the rats in our institute, and SS = the sample size.

### 2.4. Experimental Design

Twenty-four male Wistar rats were allocated to three experimental groups of eight animals each. The control group (C) received tap water ad libitum for two months. The HSL 6% group received a 6% HSL infusion ad libitum for two months. The HSL ± 6% group was given the same infusion for two months, followed by tap water for an additional two months. Throughout the experimental period, the animals were housed under controlled conditions consisting of a 12 h light/12 h dark cycle, an ambient temperature of 18–26 °C, and 40–70% relative humidity. Standard rodent chow was supplied ad libitum and contained 23% crude protein, 4.5% crude fat, 6% crude fiber, 8% ash, and 2.5% minerals (LabDiet 5008; PMI Nutrition International, Richmond, IN, USA). The protocol was approved by the Laboratory Animal Care Committee of the National Institute of Cardiology Ignacio Chávez under approval number INC/CICUAL/009/2023, and all procedures were conducted in accordance with institutional animal care guidelines. At the end of the treatment period, SBP was measured by plethysmography using a Narco Bio-Systems system (Houston, TX, USA) before euthanasia. Signals were recorded and processed with SIEVART software, version 0.1, and five measurements were obtained from each rat. The animals were then anesthetized with sodium pentobarbital at 60 mg/kg body weight. Before euthanasia, heparin was administered at 1000 U/kg body weight using a 1000 U/mL solution.

### 2.5. Isolated Langendorff Heart Model Under I/R

The heart was rapidly excised following thoracotomy and immediately immersed in ice-cold Krebs–Henseleit solution to induce cardiac arrest and minimize ischemic preconditioning. The ascending aorta was then cannulated without delay, and the organ was connected to a retrograde perfusion apparatus. Mechanical activity was maintained with Krebs–Henseleit buffer containing, in mM, 120 NaCl, 23.4 NaHCO_3_, 4.8 KCl, 1.2 KH_2_PO_4_, 0.86 MgSO_4_, 1.25 CaCl_2_, and 11.0 glucose. The solution was adjusted to pH 7.4, maintained at 37 °C, and continuously gassed with 95% O_2_ and 5% CO_2_.

A 30 min equilibration period was allowed before the experimental protocol. During this period, the coronary flow was set at 25 mL/min for the first 5 min and subsequently reduced to 14 mL/min for the remaining 25 min. Following equilibration, the perfusion flow was adjusted to 13 mL/min and maintained at this rate throughout the experimental protocol. The heart rate was maintained between 312 and 324 beats/min using square-wave stimulation delivered by a Grass S44F stimulator (Grass Instruments Co., Quincy, MA, USA). The coronary flow was controlled with a peristaltic pump (Masterflex Easy-Load II, model 77200-50; Cole-Parmer Instrument Co., Vernon Hills, IL, USA).

Left intraventricular pressure (LIVP) was measured with a Grass hydropneumatic pressure transducer (Grass Instrument Co., Quincy, MA, USA) connected to a catheter fitted with a latex balloon. The balloon was advanced through the mitral valve into the left ventricular cavity and inflated to establish a diastolic pressure of 5–10 mmHg. Perfusion pressure (PP) was monitored with a second Grass hydropneumatic pressure transducer. Hearts were included in the study only when the initial PP ranged from 55 to 70 mmHg.

All hemodynamic signals were acquired using a computerized recording system (Grass Telefactor, Grass Technologies, Astro-Med, West Warwick, RI, USA) coupled to a Grass model 79D polygraph and Grass PolyView software (Astro-Med, Inc., West Warwick, RI, USA; Grass PolyView version 3.0). Cardiac mechanical performance (CMP) was calculated as the product of heart rate and left intraventricular pressure as follows: CMP = HR × LIVP.

### 2.6. Heart Homogenization

After completing the experiments on the isolated heart, the organ was removed from the Langendorff system and segmented into three parts, where the superior and apical parts were homogenized under liquid nitrogen after adding KH_2_PO_4_ (1 mL) 0.05 mM, pH 7.3, in the presence of 20 µL of antiprotease inhibitors (2 μM leupeptin, 2 μM pepstatin A, 0.1% aprotinin, and 1 mM PMSF).

### 2.7. Anatomical Changes in the Heart by a Histological Process

To evaluate the anatomical and structural changes and collagen deposition in cardiac tissue, the central part of each heart was used for histological analysis after completing the experiments in the isolated hearts. The tissue was fixed in a 10% formalin solution for 24 h, gradually dehydrated through increasing concentrations of ethanol, cleared in xylene, embedded in paraffin, and cut into 5 µm thick sections using a microtome (Leica RM212RT, Wetzlar, Germany). The paraffin sections were stained with Masson’s trichrome and Sirius Red.

The histological sections were analyzed at 12.5× magnification using a model 63300 optical microscope (Carl Zeiss, Oberkochen, Germany) equipped with a Tucsen digital camera (Tucsen Photonics Co., Ltd., Fuzhou, China; 18 megapixels) coupled to TSView 7.3.1 software. The histological sections were evaluated by an investigator blinded to the experimental groups, and the evaluation was validated using the Allred scoring system. The microscope illumination was adjusted and kept constant throughout image acquisition. Five fields per heart were analyzed.

For quantitative collagen analysis, non-overlapping myocardial fields were selected systematically from each section, avoiding tissue folds, tears, staining artifacts, large empty spaces, and areas adjacent to the tissue margins. Collagen deposition was quantified using SigmaScan Pro 5 image-analysis software (Systat Software, Inc. in San Jose, CA, USA). For each image, the total myocardial tissue area and the collagen-positive area were determined after applying a predefined color threshold. The threshold settings were established before group comparisons and maintained unchanged for all images analyzed using the same staining method. The values were expressed in arbitrary units (pixels).

### 2.8. Evaluation of Total Antioxidant Capacity (TAC)

A total of 100 μg of homogenized heart was added to a mixture that contained C_2_H_3_O_2_ at 300 mM, FeCl_3_·6H_2_O at 20 mM, 2,4,6-tris-2-pyridyl-s-triazine at 10 mM, and HCl at 40 mM, at pH 3.6 (1.5 mL, at a ratio of 10:1:1 *v*/*v*), and incubated at 37 °C for 15 min. The absorbance was measured at 593 nm [24].

### 2.9. Nuclear Factor Erythroid 2 (NrF2)

Fifty micrograms of protein from cardiac homogenates were separated by 8% SDS-PAGE and transferred onto PVDF membranes. The membranes were blocked for 1 h at room temperature with 5% skim milk prepared in Tris-buffered saline containing Tween 20 (TBS-T). The membranes were incubated overnight at 4 °C with a monoclonal anti-phospho-Nrf2 (Ser40) antibody diluted to 1:1000 (SAB5701902-100UL; Sigma-Aldrich, St. Louis, MO, USA). After washing with TBS-T, the membranes were incubated with the appropriate secondary antibody diluted to 1:10,000 for 3 h at 4 °C. Protein bands were detected using a Bio-Rad chemiluminescent detection reagent and visualized using a ChemiDoc imaging system (Bio-Rad Laboratories, Inc., Hercules, CA, USA).

β-actin was used as the loading control and was detected using an anti-β-actin antibody diluted to 1:500 (sc-81178; Santa Cruz Biotechnology, Inc., Santa Cruz, CA, USA). After washing with TBS-T, the membranes were incubated with the appropriate secondary antibody diluted to 1:10,000 for 3 h at 4 °C. Band intensity was quantified by densitometry using a GS-800 densitometer (Bio-Rad Laboratories, Inc. in Hercules, CA, USA) and Quantity One 1-D Analysis Software, version 4.6.8 (Bio-Rad Laboratories, Inc., Hercules, CA, USA). Phospho-Nrf2 densitometric values were normalized to the corresponding β-actin signal and expressed as arbitrary units (AU).

### 2.10. Superoxide Anion Detection

The O_2_^−^ in heart homogenates was assessed by monitoring the irreversible oxidation of epinephrine to adrenochrome. Briefly, 50 μg of homogenate protein was mixed with 2 mL of 50 mM glycine buffer, adjusted to pH 10.2, followed by the addition of 50 μL of 60 mM epinephrine. The reaction was carried out at 30 °C, and the increase in absorbance at 480 nm was recorded for 6 min. O_2_^−^ was calculated using an extinction coefficient of 4.0 mM^−1^ cm^−1^ [25].

### 2.11. Statistical Analysis

Cardiac mechanical work was analyzed using a two-way repeated measures ANOVA, considering the experimental group as the between-subjects factor, time as the within-subjects factor, and the group × time interaction. Each heart was considered the experimental unit and the repeated measures factor. The stabilization and reperfusion periods were analyzed separately. Measurements during global ischemia were not subjected to inferential analysis because cardiac work was zero. Greenhouse–Geisser correction was applied to the time and group × time effects. Where appropriate, pairwise multiple comparisons were adjusted using the Holm method. Analyses of the adrenochrome densitometry for the Western blot and heart imaging, as well as general characteristics, were performed using one-way ANOVA and Tukey’s post hoc test. Data are presented as mean ± SE. Analyses and graphs were performed using Sigma Plot, version 15 (Systat Software Inc., San Jose, CA, USA). A *p*-value < 0.05 was considered statistically significant.

## 3. Results

### 3.1. Contents of Some Antioxidants Provided by HSL 6% Infusion and General Groups

Table 1 shows that the 6% HSL infusion provides diverse antioxidants, such as flavanols, polyphenols, anthocyanins, and vitamin C. Based on the mean daily fluid consumption and mean body weight of each experimental group, the estimated intake in the 6% HSL groups was 24.82 mg/kg/day of cyanidin-3-glucoside, 5.79 mg/kg/day of quercetin, 3.23 mmol/kg/day of total polyphenols, and 0.229 mmol/kg/day of vitamin C, equivalent to 40.40 mg/kg/day of ascorbic acid. In the HSL ± 6% group, the estimated intake during the HSL exposure period was 27.38 mg/kg/day of cyanidin-3-glucoside, 6.38 mg/kg/day of quercetin, 3.56 mmol/kg/day of total polyphenols, and 0.253 mmol/kg/day of vitamin C, equivalent to 44.57 mg/kg/day of ascorbic acid. During the washout period, this group did not receive HSL-derived analytes.

Table 2 shows the physiological variables of the animals before they were euthanized, where only the SBP of the C and HSL ± 6% groups in comparison with the HSL 6% group showed a significant decrease (*p* ≤ 0.03).

### 3.2. Structural Changes in the Heart

**Figure 1:**
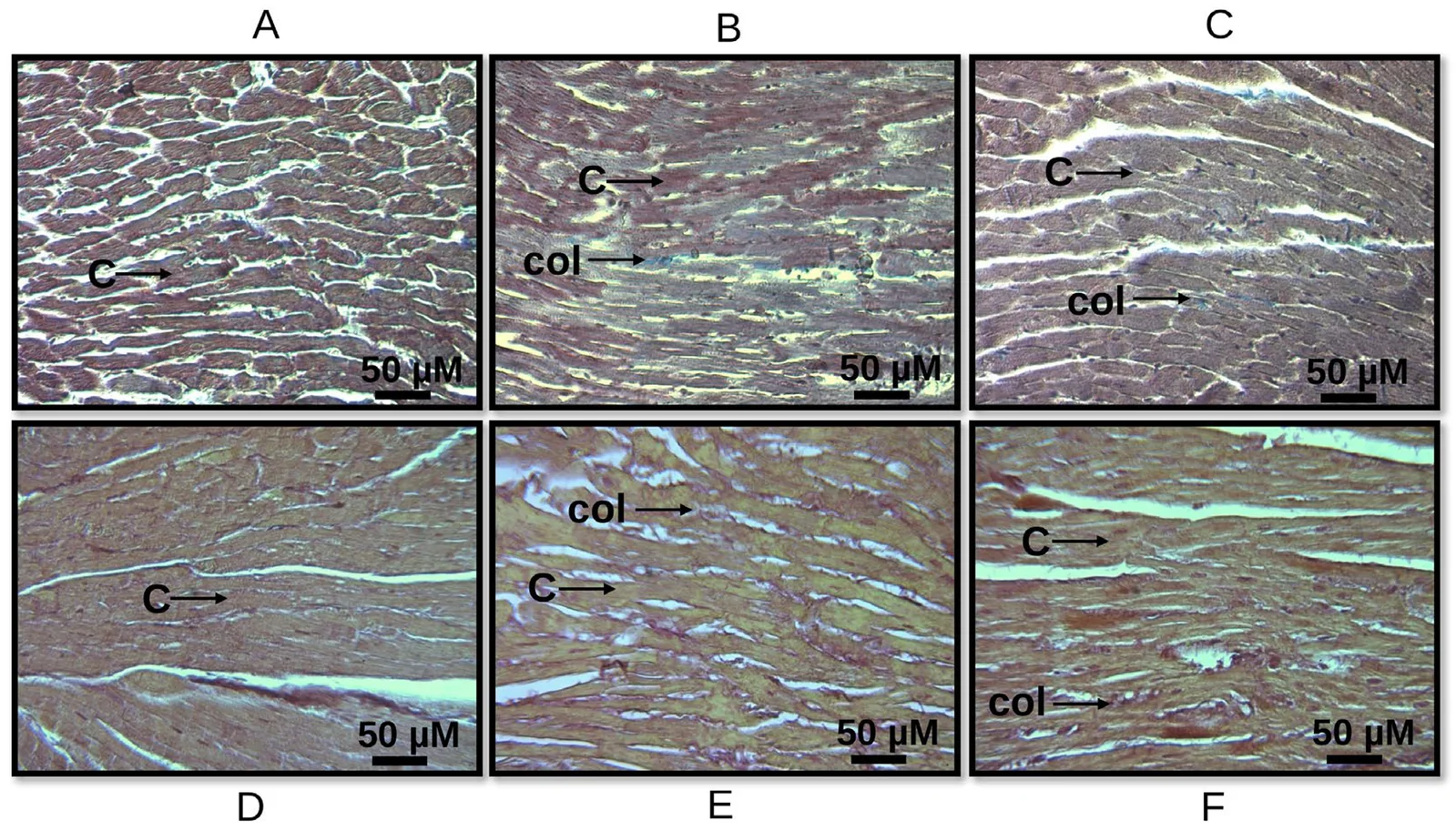
Histological evaluations of myocardial tissue using specific collagen stains. The upper panels (**A**–**C**) show sections stained with Masson’s Trichrome of the 3 experimental groups; A = C, B = HSL 6%, and C = HSL ± 6% groups, respectively. The structural organization of cardiomyocytes and interstitial connective tissue is evaluated. Panels (**D**–**F**) correspond to myocardial tissue sections stained with Syrian red in the same order as the experimental groups, which highlights the collagen fibers. The arrows indicate areas of collagen deposition/intermyofibrillar interstitial fibrosis, showing the differences in extracellular matrix accumulation between the experimental groups. Abbreviations: C = cardiomyocyte; col = collagen.

In the C group, the sections showed preserved myocardial tissue architecture, with cardiomyocyte bundles arranged in compact, parallel sheets, and homogeneous cell density. Severe damage, classic to ischemic stroke, was observed. Visible disorganization of the myofibrils was present, along with ample clear interstitial spaces. Specifically, Masson’s staining showed compact and homogeneous myocardial tissue (Figure 1A). The muscle fibers were well organized and aligned, with minimal areas of light blue staining between them, indicating a basal or normal level of interstitial collagen and thick collagen bands. Syrian red staining revealed a regular myocardial structure with thin reddish bands in the normal interstitial spaces (confirming the absence of pathological fibrosis) (Figure 1D). However, marked post-ischemic reactive fibrosis, tissue edema, and detachment of muscle bundles were evident. The tissue lost its usual elastic and compact continuity.

In the 6% HSL group, the histopathology showed increased structural damage and remodeling. Specifically, Masson’s staining revealed a loss of compact tissue continuity, i.e., areas of separation or elongation between fibers. Lighter, bluish bands or patches appeared in the interstitial space, suggesting cellular disorganization and increased collagen deposition (interstitial fibrosis) (Figure 1B). Syrian red staining showed an increased thickness and intensity of the red lines between muscle fascicles. Muscle fibers appeared slightly more separated due to the accumulation of extracellular matrix, resulting in perivascular or interstitial fibrosis, where bright red collagen surrounds and separates groups of cardiomyocytes (Figure 1E).

Finally, in the HSL ± 6% group, the morphological description of the tissue shows an intermediate structural organization between the two other experimental groups. Although subtle separations between muscle fascicles persist, the architecture of the muscle fibers appears slightly more compact, with the fibers partially regaining linear arrangement and losing compact packing (Figure 1C). Regarding Syrian red staining—which is specific for type I and III collagen—when staining with a bright red or deep pink, while the muscle cells took on a yellowish/ochre hue, marked tissue fragmentation and critical widening of the interfascicular spaces was demonstrated, revealing a significantly thinner and more restricted collagen banding pattern compared to the massive and dense infiltration observed in the HSL 6% group (Figure 1F).

**Figure 2:**
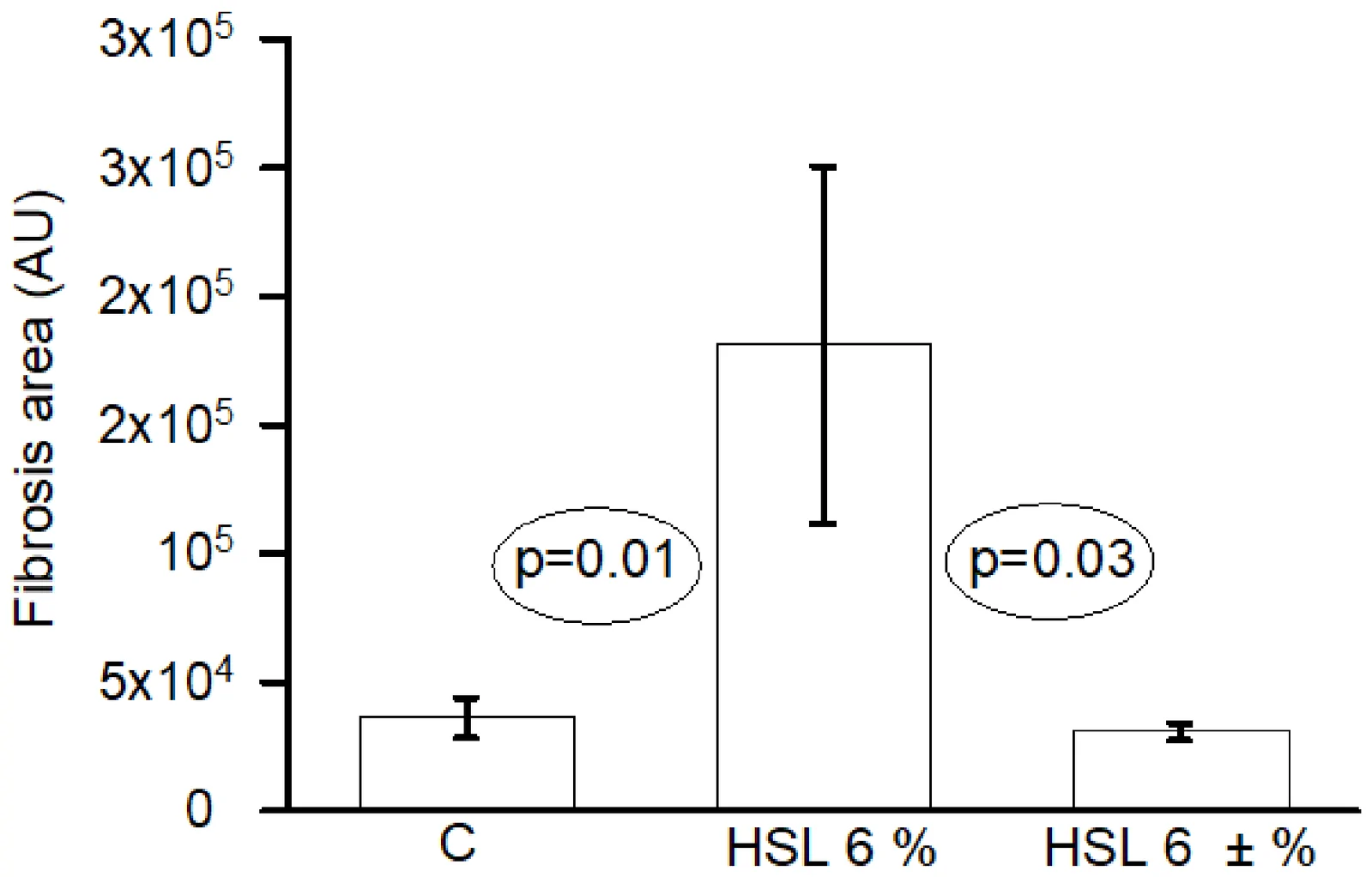
Quantification of the fibrosis area in the different experimental groups. The fibrosis area is expressed in arbitrary units (AU).

In addition, the densitometric analyses of the areas of fibrosis that were evidenced in the experimental groups by the histological process show that groups C and HSL ± 6% demonstrated a significant reduction (*p* = 0.01 and *p* = 0.03, Figure 2) in comparison with the HSL 6% group.

### 3.3. Myocardial Function and Hemodynamics

#### 3.3.1. Cardiac Mechanical Performance (CMP)

**Figure 3:**
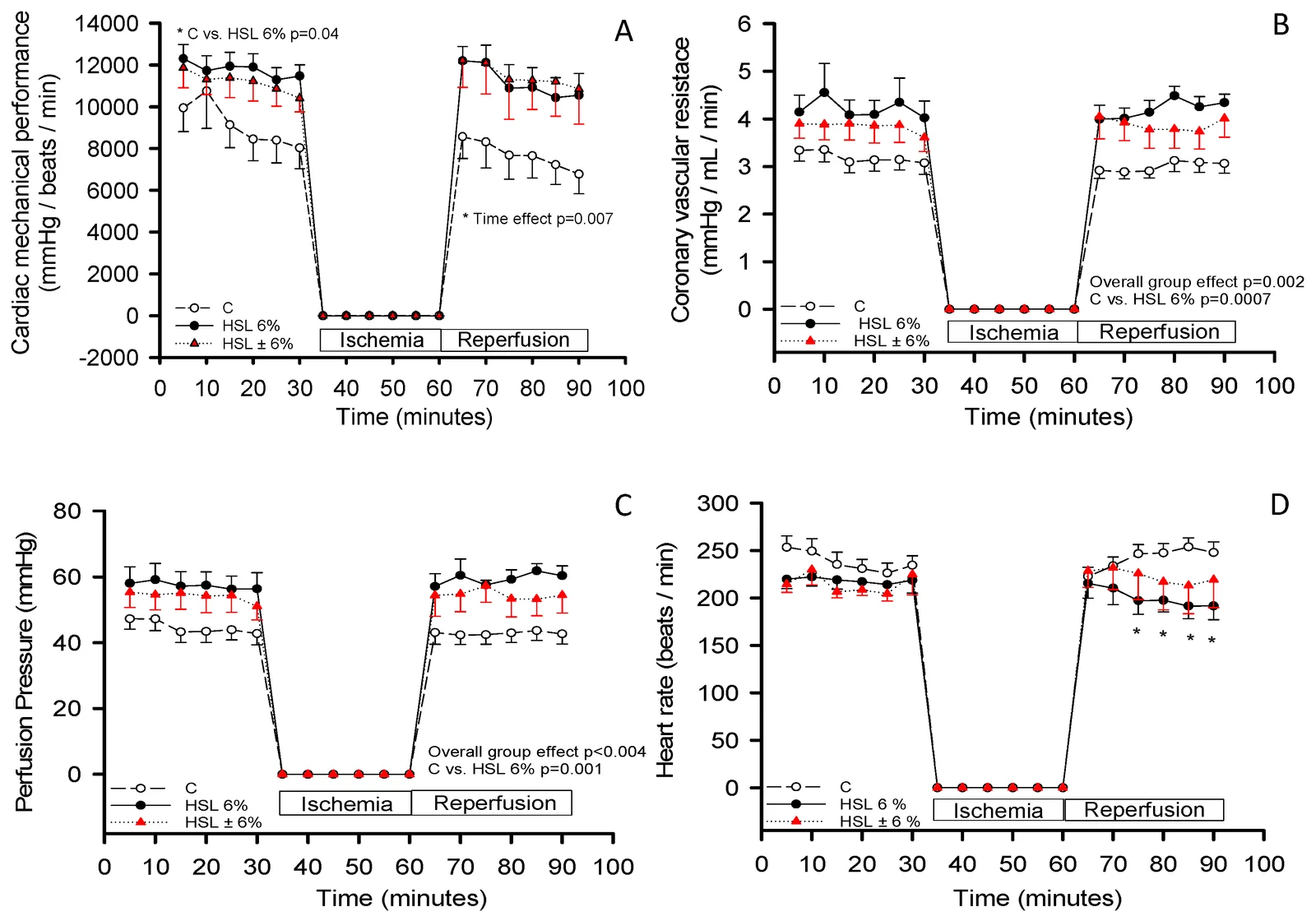
Hemodynamic variables during stabilization and I/R. In panel (**A**), a significant time effect was observed during stabilization (*p* = 0.001) and during reperfusion (*p* = 0.001). No significant differences among groups were detected. In panel (**B**), an overall group effect was detected during reperfusion (*p* = 0.003), with a higher CVR in the HSL 6% group vs. the C group (Holm-adjusted *p* = 0.0007). In panel (**C**), a significant time effect was observed during stabilization (*p* = 0.004). PP was higher in HSL 6% than in C during reperfusion (Holm-adjusted *p* = 0.0012; overall group effect, *p* = 0.005). In panel (**D**), a significant group × time interaction was observed during reperfusion (*p* = 0.010), and the HR was lower in HSL 6% than in C at 75, 80, 85, and 90 min (* Holm-adjusted *p* = 0.0389, 0.0175, 0.0055, and 0.0235, respectively). No significant differences involving the HSL ± 6% group were detected vs. the C group. Data are expressed as mean ± SE (*n* = 8). Abbreviations: C = control; HSL = *Hibiscus sabdariffa* Linnaeus. Table S1: Statistical analysis of Figure 3.

During the stabilization period, the mean CMP was 10,032.08 ± 783.60 mmHg·beats/min in the C group; for the HSL 6% group, it was 11,872.19 ± 934.25 mmHg·beats/min; and for the HSL ± 6% group, 11,180.35 ± 823.46 mmHg·beats/min. The repeated measurement analysis showed a significant effect of time [F (2.09, 43.89) = 7.69, *p* = 0.001], whereas neither the overall group effect [F (2, 21) = 1.28, *p* = 0.298] nor the group × time interaction [F (4.18, 43.89) = 2.29, *p* = 0.072] was significant. In the C and HSL ± 6% groups, CMP remained relatively stable throughout stabilization, whereas in the HSL 6% group, it showed a progressive increase toward the end of the period. No significant differences were detected among the experimental groups during stabilization. During reperfusion, the mean CMP was 8468.41 ± 749.84 mmHg·beats/min in the C group. In the 6% HSL group, it was 10,787.27 ± 759.70 mmHg·beats/min, and in the HSL ± 6% group, it was 11,490.00 ± 1492.15 mmHg·beats/min. The CMP changed significantly over time [F (3.44, 72.32) = 5.54, *p* = 0.001]. However, neither the overall group effect [F (2, 21) = 2.70, *p* = 0.090] nor the group × time interaction [F (6.89, 72.32) = 0.15, *p* = 0.992] was significant. Thus, although the HSL-treated groups showed numerically higher CMP values than the C group during reperfusion, these differences were not statistically significant (Figure 3A).

#### 3.3.2. Coronary Vascular Resistance (CVR)

During the pre-ischemic period, the mean CVR was 3.19 ± 0.22 mmHg/mL/min in the C group, 4.21 ± 0.38 mmHg/mL/min in the HSL 6% group, and 3.84 ± 0.33 mmHg/mL/min in the HSL ± 6% group. Also, no significant effect of time was observed [F (1.82, 41.92) = 2.48, *p* = 0.101] between all groups. However, the overall group effect [F (2, 23) = 3.03, *p* = 0.068] and the group × time interaction [F (3.65, 41.92) = 0.63, *p* = 0.627] did not show a statistically significant modification. Therefore, although the HSL 6% group showed numerically higher CVR values than the other groups during stabilization, no significant differences were established among the experimental groups. However, in the reperfusion period, the mean CVR was 3.00 ± 0.17 mmHg/mL/min in the C group, 4.21 ± 0.20 mmHg/mL/min in the HSL 6% group, and 3.88 ± 0.37 mmHg/mL/min in the HSL ± 6% group. The overall between-groups effect was statistically significant [F (2, 23) = 7.64, *p* = 0.003], whereas neither the time effect [F (2.36, 54.22) = 1.81, *p* = 0.167] nor the group × time interaction [F (4.72, 54.22) = 1.21, *p* = 0.317] was significant. Holm-adjusted comparisons showed that CVR was significantly higher in the HSL 6% group than in the C group (adjusted *p* = 0.0007). In contrast, the HSL ± 6% group did not show significant differences with the HSL 6% and C groups (Figure 3B).

#### 3.3.3. Perfusion Pressure (PP)

The mean of the PP during the stabilization period (pre-ischemic) was 44.65 ± 3.14 mmHg in the C group, 57.41 ± 4.41 mmHg in the HSL 6% group, and 54.12 ± 4.68 mmHg in the HSL ± 6% group. A significant effect of time was observed between all groups [F (3.35, 77.01) = 4.48, *p* = 0.004]. However, the overall group effect [F (2, 23) = 2.97, *p* = 0.071] and the group × time interaction [F (6.70, 77.01) = 0.86, *p* = 0.539] were not significant. Therefore, although PP was numerically higher in both HSL 6% and HSL ± 6% groups vs. the C group, these differences did not reach statistical significance. In the reperfusion period, the mean of the PP was 42.87 ± 2.98 mmHg in the C group, 59.41 ± 2.25 mmHg in the HSL 6% group, and 54.58 ± 5.28 mmHg in the HSL ± 6% group. A significant overall group effect was observed [F (2, 23) = 6.76, *p* = 0.005]. In contrast, neither the time effect [F (3.06, 70.45) = 0.27, *p* = 0.853] nor the group × time interaction [F (6.13, 70.45) = 0.72, *p* = 0.635] was significant. Holm-adjusted comparisons showed that PP was significantly higher in the HSL 6% group than in the C group (adjusted *p* = 0.0012). In contrast, the HSL ± 6% group did not show significant differences from the HSL 6% and C groups (Figure 3C).

#### 3.3.4. Heart Rate (HT)

The mean of the HR was 238. 36 ± 9.66 beats/min in the C group, 218.51 ± 9.58 beats/min in the HSL 6% group, and 214.89 ± 7.91 beats/min in the HSL ± 6% group. No significant effect of time was observed [F (2.94, 67.60) = 2.74, *p* = 0.051]. However, the overall group effect [F (2, 23) = 1.88, *p* = 0.176] and the group × time interaction [F (5.88, 67.60) = 0.73, *p* = 0.626] were not statistically significant. Thus, although both HSL-exposed groups showed numerically lower HR values than the C group, no significant differences among groups were detected during stabilization. During the reperfusion period, the mean of the HR was 242.05 ± 9.00 beats/min in the C group, 200.67 ± 12.65 beats/min in the HSL 6% group, and 222.61 ± 25.79 beats/min in the HSL ± 6% group. However, a significant group × time interaction was observed [F (5.14, 59.09) = 3.31, *p* = 0.010], indicating that the temporal HR response during reperfusion differed among groups. Neither the overall group effect [F (2, 23) = 1.99, *p* = 0.159] nor the time effect [F (2.57, 59.09) = 0.20, *p* = 0.868] was significant. Holm-adjusted comparisons showed that the HR in the HSL 6% group was significantly lower than in the C group at 75 min (197.30 vs. 246.58 beats/min, adjusted *p* = 0.0389), 80 min (197.64 vs. 247.53 beats/min, adjusted *p* = 0.0175), 85 min (191.54 vs. 253.79 beats/min, adjusted *p* = 0.0055), and 90 min (191.64 vs. 247.83 beats/min, adjusted *p* = 0.0235). In contrast, the HSL ± 6% group did not show significant differences from the HSL 6% and C groups at the evaluated reperfusion time points (Figure 3D).

### 3.4. Electrocardiograms (ECG)

**Figure 4:**
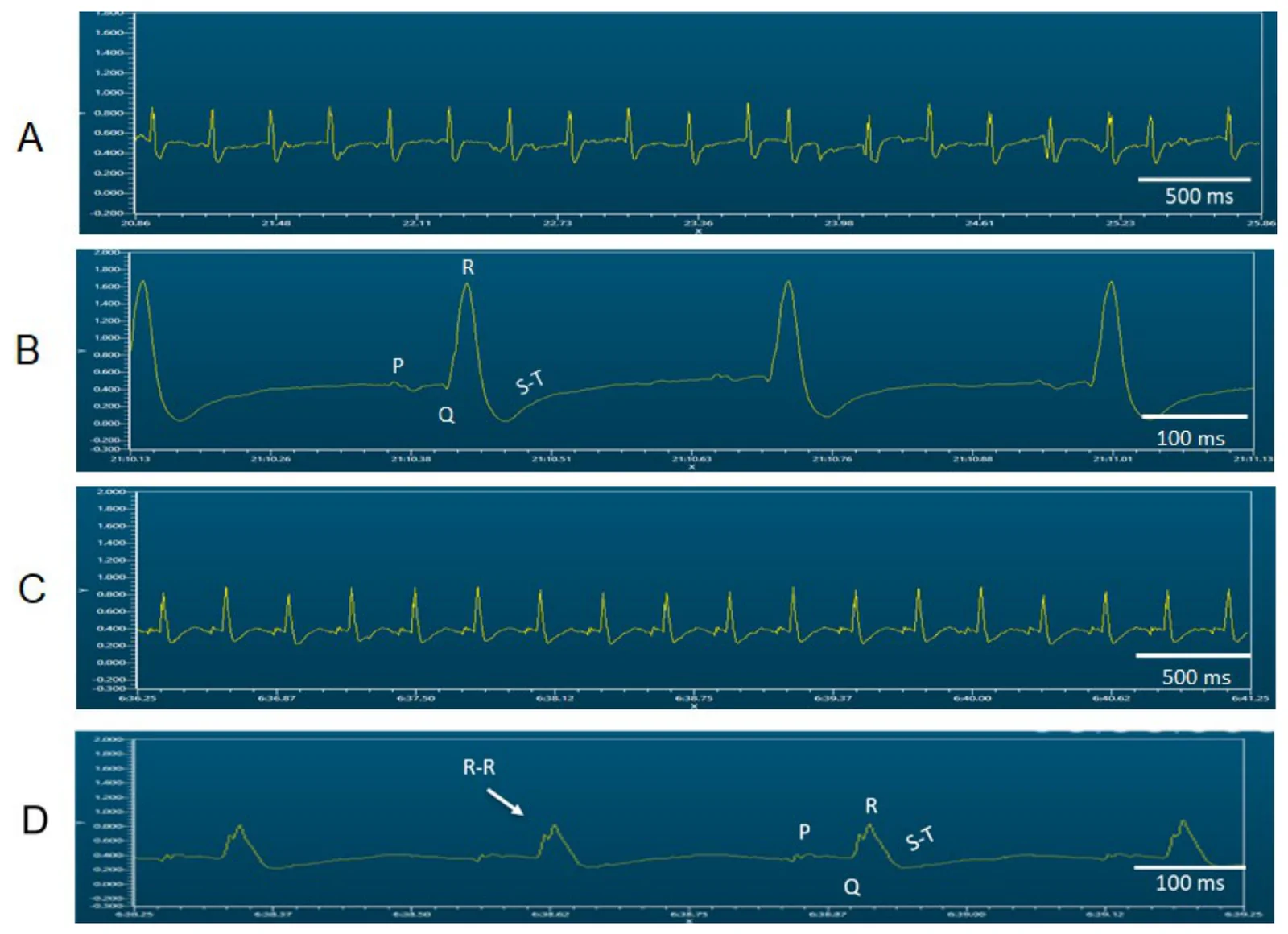

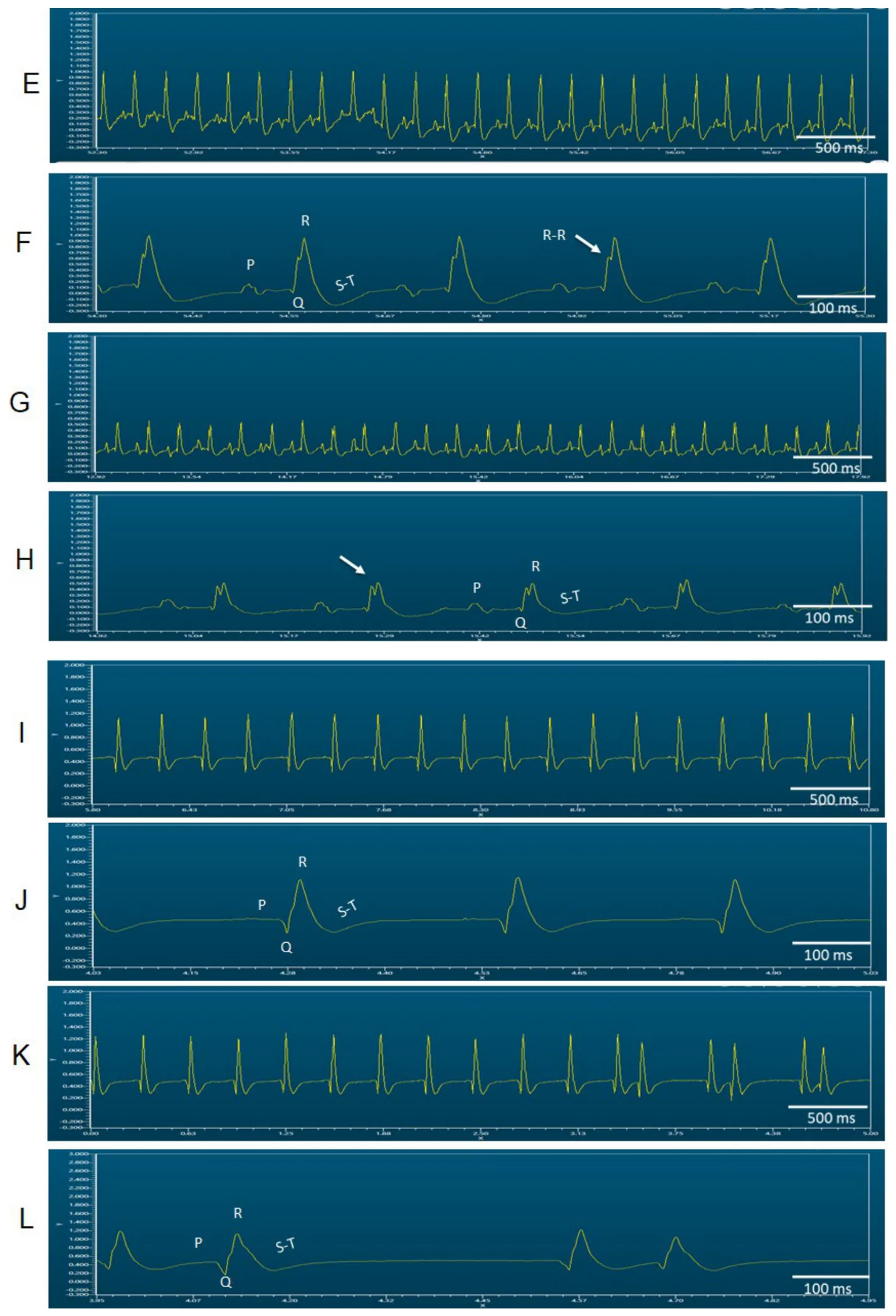
Representative ECG recordings of isolated rat hearts under baseline conditions and after I/R. Tracings at 500 ms and 100 ms (enlarged view) of the C group show a relatively regular electrical pattern during stabilization (**A**,**B**) and a premature-appearing widened complex post-I/R (**C**,**D**). The HSL 6% group exhibited greater variability in rhythm regularity and complex amplitude at baseline, with a premature-appearing complex (**E**,**F**) and irregular and distorted ventricular complexes after I/R (**G**,**H**). The HSL ± 6% group shows a relatively homogeneous and regular pattern under baseline conditions (**I**,**J**) but shows prolonged pauses and changes in complex morphology during reperfusion (**K**,**L**). Arrows indicate premature-appearing complexes (**D**,**F**) or widened and fragmented ventricular complexes (**H**). These tracings are presented as illustrative observations only and were not quantitatively analyzed for arrhythmia incidence, duration, severity, or overall arrhythmic burden.

Variations in rhythm and waveform morphology were observed among the experimental groups; however, these recordings were not subjected to quantitative analysis. Therefore, the tracings should be interpreted as illustrative examples only, and no conclusions regarding arrhythmia incidence, duration, severity, or overall arrhythmic burden can be established from these observations. Group C presented a regular ECG pattern using the Langendorff method. Figure 4A shows cyclic complexes with constant intervals and homogeneous amplitudes at 500 ms. In Figure 4B, the tracing, magnified to 100 ms, allowed observations of detailed, well-defined waves, where the P wave (atrial depolarization) and the QRS complex with a narrow, tall R wave (ventricular depolarization), and the S-T segment showed no evident alterations. The panels after the I/R test (Figure 4C,D) showed loss of regularity at 500 ms, evidenced by variations in HR and complex amplitude. The 100 ms magnification, highlighted with an arrow, shows a premature-appearing and widened complex.

In the ECG of rat hearts that received the HSL 6% infusion, baseline electrical irregularities were visually observed in Figure 4E,F. Figure 4E at 500 ms showed notable variations in amplitude and apparently shorter intervals between complexes before damage was induced. Similarly, in Figure 4F at 100 ms, a premature-appearing complex marked R-R is clearly visible. These findings are presented as illustrative observations and do not establish a predisposition to arrhythmias. Furthermore, after I/R, Figure 4G at 500 ms showed a marked loss of cyclic regularity and a pronounced reduction in the amplitude of the QRS complexes. Meanwhile, in Figure 4H at 100 ms, the white arrow points to distorted and widened ventricular complexes. These observations do not allow conclusions regarding lethal ventricular arrhythmias or the extent of myocardial damage.

On the other hand, Figure 4I shows a representative ECG of the HSL ± 6% group at 500 ms, where a markedly more homogeneous cyclical tracing with stable R-wave amplitudes was observed, similar to the C group. In the magnification of Figure 4J at 100 ms, the P, QRS waves, as well as the S-T segment, appear clear and well-defined; this is unlike the ECG in Figure 4F, where no premature-appearing basal complexes were visually observed in this representative tracing. During the reperfusion period, Figure 4K at 500 ms shows that, despite the 31 min ischemic insult, the heart maintained well-defined QRS complexes and a relatively regular pattern for most of the tracing, unlike the marked irregularity observed in Figure 4G. Finally, Figure 4L at 100 ms shows notable pauses and prolonged intervals between complexes, and the aberrant polymorphic, wide, and fragmented complexes recorded in Figure 4H were not observed. However, these qualitative findings do not demonstrate the absence of polymorphic ventricular tachycardia or ventricular premature beats. These results suggest that visual differences in electrical patterns were present under baseline and reperfusion conditions. Because the ECG events were not quantified, no conclusions can be drawn regarding restoration of membrane potential, suppression of basal ectopia, recovery of conduction properties, ventricular synchrony, cell density, automaticity, or electrophysiological function.

Overall, these representative tracings illustrate differences in rhythm regularity, waveform morphology, and complex amplitude among the experimental groups and between baseline and reperfusion conditions. However, because the incidence, duration, and severity of ECG events were not quantified, these observations should be interpreted only as illustrative and do not establish differences in arrhythmia burden.

**Figure 5:**
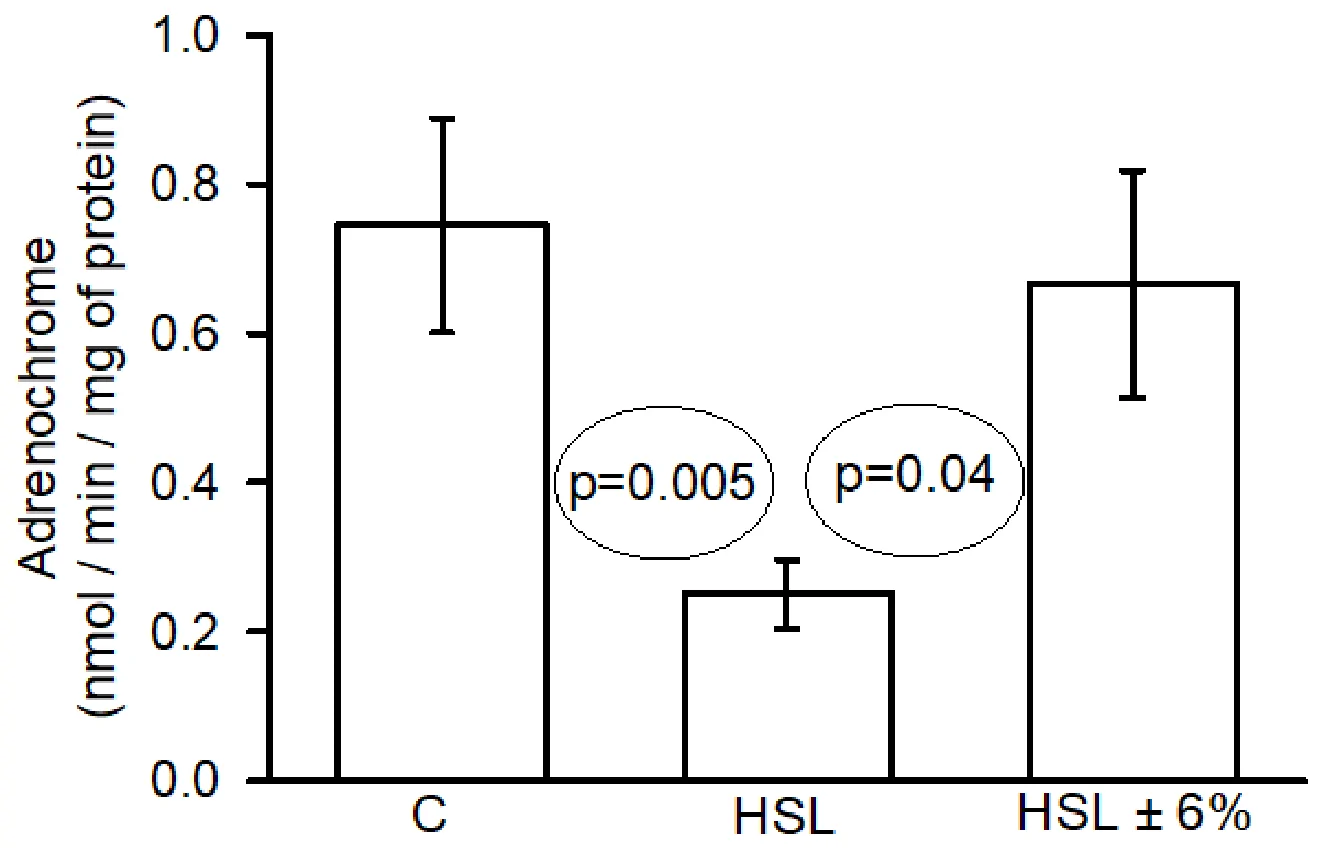
Adrenochrome formation, used as an indirect indicator of homogenized O_2_^−^ production in the heart. Data are presented as mean ± SE (*n* = 8).

Figure 5 shows the O_2_^−^ production in cardiac homogenates, estimated through adrenochrome formation. O_2_^−^ production was significantly lower in the HSL 6% group than in the C group (*p* = 0.005). In the HSL 6% group, the adrenochrome was significantly lower vs. the HSL ± 6% group (*p* = 0.04). The HSL ± 6% group did not show statistically significant differences with the C group.

**Figure 6:**
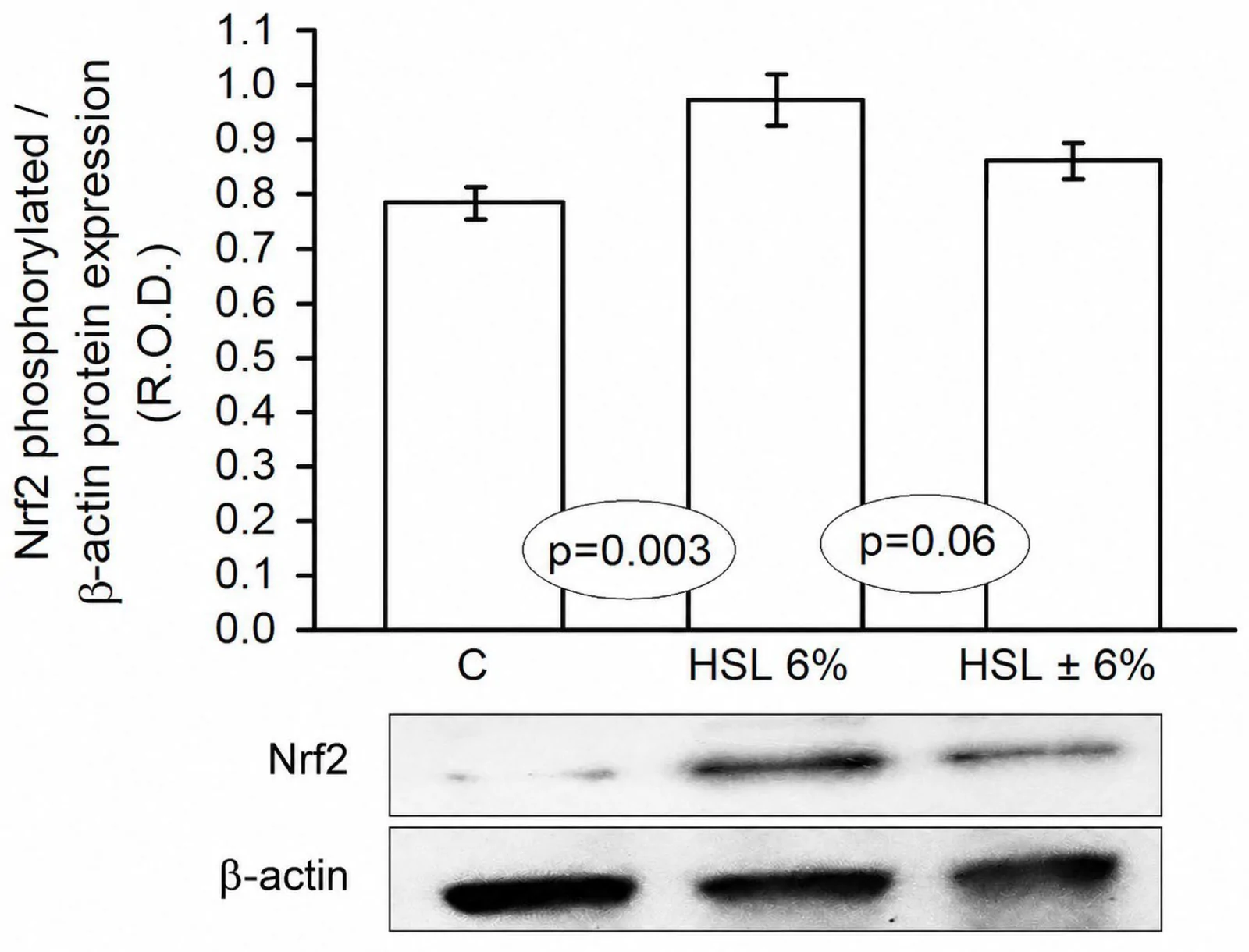
Representative Western blot images that represent the expression of Nrf2/β-actin in the heart homogenate in the experimental groups. Data are presented as mean ± SE (*n* = 8).

Figure 6 shows the Nrf2 phosphorylation/β-actin ratio expression, in which there was an increase in the HSL 6% group in comparison with the C group (*p* = 0.003). The HSL ± 6% group also showed higher values than the C group; however, this difference did not reach statistical significance (*p* = 0.06).

## 4. Discussion

In the present study, we evaluated whether chronic and excessive ingestion of a 6% HSL infusion for two months compromised the structural, mechanical, and hemodynamic performance of the myocardium during I/R in hearts of healthy Wistar rats. Chronic exposure to 6% HSL is a rat experimental model that has been previously characterized by our group as producing systemic and renal alterations associated with antioxidant overload and probably RS [7,8,9,10]. In the present study, the increase in TAC and phospho-Nrf2 expression, together with reduced adrenochrome formation after I/R, indicate an enhanced antioxidant response and a redox environment consistent with increased reduced capacity. In this paper, we also evaluated whether the removal of the infusion could revert the damaging effects of RS associated with the ingestion of the infusion.

The heart is subjected to a period of restricted blood flow (ischemia) followed by the restoration of flow (reperfusion) in experimental models of I/R. While restoring flow is essential to save ischemic tissue, there is a reperfusion paradox that states that it triggers a secondary wave of tissue damage, microvascular dysfunction, and arrhythmias that are caused, in part, by the large amount of ROS generated during this period [25]. Each of the parameters chosen for evaluation of the damage caused by I/R in this study, including CTM, CVR, PP, and HR, provides a window into the distinct I/R pathological mechanisms [26]. These parameters do not operate in isolation; they are deeply intertwined components of a dynamic physiological loop [27].

CMP is an indicator of myocardial viability and functional recovery, and it reflects the degree to which the myocardium has suffered irreversible damage versus reversible stunning [27,28]. Our results showed that in both the HSL 6% and the HSL ± 6% groups, the CMPs were ~12,000 mmHg/beats/min. This was maintained despite the reperfusion period. In contrast, in the C group, there was a decrease of nearly ~7000. This suggests that the antioxidant overload provided by the HSL infusion could inhibit or neutralize the overproduction of ROS generated during the reperfusion period. Restoring flow and the abrupt return of oxygen that normally triggers ROS overproduction [8], which saturates the basal antioxidant system, was not observed after ingestion of the infusion. When ROS levels increased at the time of reperfusion, the tissue possibly already had a massive and abnormally high reserve of antioxidants that were accumulated during the two months of treatment [9]. These accumulated antioxidants may immediately neutralize the ROS generated by reperfusion by reacting with the excess reducing equivalents [8,9]. However, in the HSL ± 6% group, the washout period was not long enough, and the heart still showed alterations.

The CVR measures the resistance to flow within the coronary circulation. A rising CVR during reperfusion is a direct hallmark of the “no-reflow” phenomenon and determines the level of protection of the coronary vasculature [27,28]. Our results from the C group show that the CVR was constant, which suggests that the intramyocardial coronary arteries were free of external mechanical compression before ischemia occurred; however, after reperfusion, a massive contractile effect occurred, suggesting that the myocardium no longer contracted with force and ceased to compress the blood vessels during systole, eliminating internal mechanical resistance and maintaining low VR [29]. However, in the 6% HSL group, the results showed a high CRV. This suggests that antioxidant saturation altered basal cell signaling in the myocardial tissue, suggesting chronic structural remodeling of the cardiac muscle, evidenced by histological staining of the heart sections. This structural alteration can cause extrinsic mechanical compression of the coronary microvasculature, physically reducing the diameter of intramural blood vessels. As the caliber of the flow conduction pathways may be decreased, hydrodynamic resistance might be chronically elevated, which is why this group had a higher CVR, even before the recording of the ischemic period. However, once the reperfusion condition is established, the antioxidant molecules provided by the 6% HSL infusion may overexpress the antioxidant system and deplete ROS [9,10]. Consequently, the membranes of endothelial cells and smooth vascular muscle are possibly protected from the ROS generated during reperfusion, resulting in the CVR remaining at high levels since the chronic structural compression factor dominates and prevents resistance from decreasing [30].

In addition, the PP is a direct reflection of the hydrostatic force required to push fluid through the arteries of the heart. Both PP and CVR are directly connected by Ohm’s hydrodynamic law; since the flow is constant, PP behaves exactly like CVR [30,31]. Therefore, a low PP in the C group indicates that in this group, vessels are passively and pathologically relaxed, lacking active myogenic regulation due to acute cellular stunning from ischemia–reperfusion. A dramatic spike in PP during reperfusion suggests severe vasoconstriction or microvascular plugging, induced primarily by the ROS burst and cytosolic Ca^2+^ overload [32]. Our results show that a chronic intake of high doses of antioxidants for two months by drinking the 6% HSL solution may have possibly induced an RS state that altered the basal redox signaling pathways necessary for extracellular matrix homeostasis. Furthermore, it generated a structural alteration that might have induced a constant extrinsic mechanical compression on intramural coronary arteries, physically narrowing their internal diameter (vascular lumen) [10]. With the reduced caliber of the arteries, the system would require significantly more force to push perfusion fluid through them. This structural compression might be the direct reason why the PP was elevated at the beginning of the experiment and remained elevated throughout. In hemodynamic terms, the presence of the perivascular fibrosis exerts extrinsic mechanical compression on the intramural coronary arteries, decreasing the diameter of the vascular lumen and increasing resistance to hydrostatic flow [33]. The coexistence of collagen accumulation with elevated CMP, CVR, and PP suggests a relationship between myocardial fibrosis and an impaired functioning of the heart. Nevertheless, the current experimental design does not establish a temporal sequence, nor does it prove that altered ROS levels or NrF2 activation directly caused collagen deposition and the subsequent functional abnormalities. Finally, the results from the washout group (HSL ± 6%) showed that removal and reintroduction of tap water partially eliminated the antioxidant overload provided by the HSL infusion, reducing extrinsic mechanical compression in the intramural coronary arteries. As a result, the blood vessels recovered their original internal diameters.

The HR is a variable that dictates both energy supply and demand of the cardiac muscle [27]. In this sense, the C group exhibited marked bradycardia, electrical instability, and reduced CMP after reperfusion, indicating impaired post-ischemic functional recovery. Alterations in myocardial energetic status, redox regulation, or impulse generation may have contributed to these findings; however, these mechanisms were not directly evaluated in the present study. We were also not able to quantify the incidence of arrhythmia, its duration, or severity. During reperfusion, the low CVR and PP observed in the C group could indicate impaired coronary hemodynamic recovery. Endothelial viability, smooth muscle function, myogenic tone, and vasoplegia were not directly assessed; therefore, these mechanisms remain hypothetical [34]. The relative preservation of the HR in the HSL-treated groups occurred in parallel with increased reserves of antioxidants and changes in the myocardial redox environment. These findings suggest that modulation of the ROS response may have influenced the chronotropic response during reperfusion. However, the present results do not demonstrate that ROS neutralization directly preserved sinoatrial automaticity or prevented arrhythmogenic mechanisms [35].

The results obtained in the illustrative representative ECG recordings show electrical abnormalities observed in the experimental groups and could be considered contradictory, since the excess antioxidants and the lack of basal oxidants could act as a shield for protection against ROS; that is, there is normally an increase in the generation of ROS after reperfusion that may cause damage, as observed in the C group. However, in the experimental HSL groups, ROS reacted with the enormous reserve of antioxidants and reducing equivalents accumulated in the tissue instead of causing damage. The combined analysis of representative ECG recordings provides electrophysiological evidence that validates the mechanical (CMP and HR) and hemodynamic (CVR and PP) alterations. For example, in the C group, the adaptation period (0–30 min) caused a widening and distortion of the QRS complex, loss of P-wave definition, and critical alterations in the S-T segment [36]. The widening and distortion of the ventricular complexes, together with the morphological abnormalities, are compatible with impaired ventricular electrical coordination and possible conduction disturbances. The coexistence of these electrical abnormalities with reduced mechanical performance could suggest altered electromechanical integration during reperfusion [28,30]. Nevertheless, conduction velocity, ventricular synchrony, intracellular calcium handling, and excitation–contraction coupling were not directly evaluated and therefore cannot be confirmed from the present data.

In the ECG of the HSL 6% group, excess antioxidants may have acted as a metabolic shield. They neutralized the acute burst of ROS [9,10], and consequently, double deflections or notches were observed in the activation complex. The findings in the ECG, histological observations, and the decreased ROS indicate that chronic exposure to the 6% HSL infusion produced a complex myocardial response, in which increased collagen deposition was accompanied by extracellular matrix remodeling and alterations in the ECG. In contrast, in the HSL ± 6% group, the ECG suggests that the PQR and S-T complexes showed a cleaner, sharper tracing, completely free of the notches or double deflections present in the HSL 6% group. This indicates that the two months of prior HSL treatment could indicate induced lasting electrical and metabolic preconditioning. However, upon the onset of I/R, the tissue remained partially protected against ROS damage, preserving automaticity.

Additionally, HSL contains minerals such as aluminum (Al), which are potentially toxic to humans. Al is first eliminated in urine and feces, but part of the Al can be retained in the organism and accumulate. In this sense, a study of volunteers who drank an HSL infusion at 2.5% for 16 days showed a high Al content in their urine that was associated with abnormalities such as diarrhea, gastrointestinal problems, nausea, and dizziness [37]. This suggests that high concentrations of the HSL infusion could lead to an increased Al body burden and cause cardiovascular problems.

On the other hand, regarding the potential damage caused by excessive antioxidants in human health, it has been described that drug toxicity due to overdoses or drug interactions in healthy subjects and in subjects with comorbidities who chronically consume medications or supplements, whether prescribed by a physician or administered through self-medication, can occur. Although the goal of treatment is to improve physical conditions or alleviate discomfort, the use of some medications, due to drug interactions, can synergize or reduce the kinetic effect of other drugs. Studies in the literature have reported no association between antioxidant use and mortality. However, these studies did not analyze the oxidative or reducing status of the subjects who consumed them [38,39]. Therefore, the inclusion of the use of antioxidants by patients in future randomized clinical trials should be considered to allow for the analysis of oxidative or RS as causes of morbidity and mortality worldwide [40]. Many patients exhibit cardiac remodeling, and medications, along with the individual’s genetic involvement, may modulate Nrf2 [39]. In this regard, the activation of Nrf2 in the hearts of transgenic mouse models increases endogenous antioxidants and induces RS that is associated with the development of hypertrophic cardiomyopathy [10]. Over time, this progresses to diastolic dysfunction and heart failure, and the damage is attributed to excessive antioxidant signaling. Therefore, in human patients with heart failure receiving antioxidant supplementation therapy, a thorough assessment of redox homeostasis is suggested and should be performed prior to treatment [40].

## 5. Conclusions

Our study shows alterations in critical mechanical and hemodynamic cardiac function associated with the excessive ingestion of antioxidants provided by HSL infusion. These alterations became more evident during I/R. It also shows structural cardiac alterations associated with increased ingestion of the infusion. These findings indicate that a chronic high-dose intake of HSL was associated with cardiac alterations under the experimental conditions evaluated. Furthermore, the washout group showed possible partial differences in cardiac functional variables associated with withdrawal; however, these findings cannot be attributed solely to withdrawal because this group was evaluated after a longer experimental period. The underlying mechanisms triggered by the ingestion of high doses of antioxidants were not directly evaluated, and therefore, RS remains only a proposed, unconfirmed mechanism.

## 6. Study Limitations

This study has several limitations that should be considered when interpreting the findings. The use of isolated hearts from male Wistar rats subjected to ex vivo ischemia–reperfusion limits the extrapolation of the results to in vivo cardiac physiology, the other gender (female), and humans. Moreover, the 6% HSL infusion represents a high experimental exposure that may not reflect customary human consumption. Although the increased TAC, adrenochrome NrF2, and associated functional and histological alterations support the presence of an altered redox environment, RS was not comprehensively confirmed through direct measurements of reducing equivalents or redox couples. However, because these measurements were obtained from myocardial homogenates collected after I/R and direct redox couples were not measured, they do not independently establish myocardial RS or prove a direct causal relationship with the observed cardiac alterations. Therefore, myocardial RS is considered a mechanistically supported interpretation derived from the previously characterized model rather than a causal pathway definitively demonstrated by the present cardiac measurements alone. Likewise, the collagen deposition and incomplete recovery after the two-month washout period suggest persistent structural remodeling, but they do not demonstrate that these alterations are permanent, particularly because longer follow-up periods were not evaluated. Another important limitation of the experimental design is that the C and HSL 6% groups were evaluated after two months, whereas the washout group was evaluated after four months by adding the two months of HSL water consumption. Therefore, the differences observed in the washout group may reflect not only the effects of HSL withdrawal but also the longer study duration and physiological changes associated with aging. The absence of a time-matched C group maintained for four months under the same experimental conditions prevents a clear distinction between washout-related effects and those attributable to study duration. Consequently, the reductions in CMP, CVR, PP, and some electrocardiographic abnormalities in the washout group should be interpreted as a possible partial recovery associated with HSL withdrawal rather than definitive evidence of reversibility. Likewise, the persistence of collagen deposition and some functional abnormalities does not demonstrate that the damage is permanent. Future studies should include age- and time-matched C groups, as well as longitudinal assessments at different time points after withdrawal, to more accurately determine the reversibility and temporal evolution of the observed changes.

## Figures and Tables

**Table 1:** Antioxidant molecules provided by HSL 6% infusion.

Analytes of the HSL 6% Infusion	
Cyanidin-3-glucoside (mg/L)	466.30 ± 58.02
Quercetin (mg/L)	108.73 ± 31.76
Polyphenols (mmol/L)	60.65 ± 21.67
Vitamin C (mM/L)	4.31 ± 0.11

**Table 2:** General characteristics in experimental groups: * C and HSL ± 6% vs. HSL 6%; *p* ≤ 0.03. Abbreviations: SBP = systolic blood pressure; TAC = total antioxidant capacity.

Variables	C	HSL 6%	HSL ± 6%
Water consumption (mL/24 h)	17.5 ± 1.5	23.5 ± 2.2	24.8 ± 2.9
Body weight (g)	456.5 ± 10.3	441.5 ± 11.3	422.4. ± 20.4
SBP (mmHg)	135 ± 1.6	153.6 ± 3.7 *	139.7 ± 2.7 *
TAC (nM)	83.07 ± 5.52	97.40 ± 2.07 *	89.67 ± 2.53 *

## Data Availability

The raw data supporting the conclusions of this article will be made available by the authors on request.

## Supplementary Materials

The following supporting information can be downloaded at: https://www.mdpi.com/article/10.3390/toxics14080671/s1, Table S1: Statistical analysis of Figure 3.

## Abbreviations

HSL	*Hibiscus sabdariffa* Linnaeus
OS	Oxidative stress
ROS	Reactive oxygen species
RS	Reductive stress
TAC	Total antioxidant capacity
SBP	Systolic blood pressure
ECM	Extracellular matrix
I/R	Ischemia–reperfusion
CV	Conduction velocity
LIVP	Left intraventricular pressure
PP	Perfusion pressure
CMP	Cardiac Mechanical Performance
CVR	Coronary Vascular Resistance
HR	Heart Rate
