# Supplementary material for: Physiological Changes During Ischemia–Reperfusion Indicating Myocardial Damage Induced by Chronic and Excessive Consumption of Hibiscus sabdariffa L
Source: Toxics. 2026 Jul 29;14(8):671. doi: 10.3390/toxics14080671 (PMC13517620; doi:10.3390/toxics14080671)
Supplement: Supplementary file 1 [file toxics-14-00671-s001.zip › toxics-4447550-supplementary.pdf]

Two-way repeated-measures ANOVA										
Experimental groups	Variable	Phase	Effect	F	df1	df2	GG epsilon	p value	Significant	Interpretation
C; HSL 6%; HSL ± 6%	CMP	Stabilization	Group	1.2819	2.0000	21.0000		0.298359105	No	No overall group difference.
C; HSL 6%; HSL ± 6%	CMP	Stabilization	Time	7.6892	5.0000	105.0000	0.4180	0.001185934	Yes	Values changed across time.
C; HSL 6%; HSL ± 6%	CMP	Stabilization	Group × time	2.2861	10.0000	105.0000	0.4180	0.072604251	No	Temporal pattern did not differ among groups.
C; HSL 6%; HSL ± 6%	CMP	Reperfusion	Group	2.7018	2.0000	21.0000		0.09033054	No	No overall group difference.
C; HSL 6%; HSL ± 6%	CMP	Reperfusion	Time	5.5384	5.0000	105.0000	0.6888	0.001083551	Yes	Values changed across time.
C; HSL 6%; HSL ± 6%	CMP	Reperfusion	Group × time	0.1555	10.0000	105.0000	0.6888	0.992241302	No	Temporal pattern did not differ among groups.
C; HSL 6%; HSL ± 6%	CVR	Stabilization	Group	3.0265	2.0000	23.0000		0.06810504	No	No overall group difference.
C; HSL 6%; HSL ± 6%	CVR	Stabilization	Time	2.4776	5.0000	115.0000	0.3645	0.10071247	No	No significant temporal change.
C; HSL 6%; HSL ± 6%	CVR	Stabilization	Group × time	0.6346	10.0000	115.0000	0.3645	0.626553513	No	Temporal pattern did not differ among groups.
C; HSL 6%; HSL ± 6%	CVR	Reperfusion	Group	7.6396	2.0000	23.0000		0.002856284	Yes	Overall difference among experimental groups.
C; HSL 6%; HSL ± 6%	CVR	Reperfusion	Time	1.8091	5.0000	115.0000	0.4715	0.167459456	No	No significant temporal change.
C; HSL 6%; HSL ± 6%	CVR	Reperfusion	Group × time	1.2105	10.0000	115.0000	0.4715	0.316961305	No	Temporal pattern did not differ among groups.
C; HSL 6%; HSL ± 6%	PP	Stabilization	Group	2.9727	2.0000	23.0000		0.071073444	No	No overall group difference.
C; HSL 6%; HSL ± 6%	PP	Stabilization	Time	4.4822	5.0000	115.0000	0.6697	0.004425117	Yes	Values changed across time.
C; HSL 6%; HSL ± 6%	PP	Stabilization	Group × time	0.8587	10.0000	115.0000	0.6697	0.538961313	No	Temporal pattern did not differ among groups.
C; HSL 6%; HSL ± 6%	PP	Reperfusion	Group	6.7567	2.0000	23.0000		0.004916588	Yes	Overall difference among experimental groups.
C; HSL 6%; HSL ± 6%	PP	Reperfusion	Time	0.2673	5.0000	115.0000	0.6126	0.852612866	No	No significant temporal change.
C; HSL 6%; HSL ± 6%	PP	Reperfusion	Group × time	0.7229	10.0000	115.0000	0.6126	0.635270654	No	Temporal pattern did not differ among groups.
C; HSL 6%; HSL ± 6%	HR	Stabilization	Group	1.8768	2.0000	23.0000		0.175780584	No	No overall group difference.
C; HSL 6%; HSL ± 6%	HR	Stabilization	Time	2.7379	5.0000	115.0000	0.5879	0.051227431	No	No significant temporal change.
C; HSL 6%; HSL ± 6%	HR	Stabilization	Group × time	0.7278	10.0000	115.0000	0.5879	0.626114425	No	Temporal pattern did not differ among groups.
C; HSL 6%; HSL ± 6%	HR	Reperfusion	Group	1.9949	2.0000	23.0000		0.158882028	No	No overall group difference.
C; HSL 6%; HSL ± 6%	HR	Reperfusion	Time	0.2013	5.0000	115.0000	0.5139	0.867973387	No	No significant temporal change.
C; HSL 6%; HSL ± 6%	HR	Reperfusion	Group × time	3.3106	10.0000	115.0000	0.5139	0.009977759	Yes	Groups followed different temporal patterns.

Table S1. Statistical analysis of figure 3.
